# Effects of Different Doses of Exercise on Inflammation Markers Among Adolescents With Overweight/Obesity: HEPAFIT Study

**DOI:** 10.1210/clinem/dgac021

**Published:** 2022-01-17

**Authors:** Robinson Ramírez-Vélez, Antonio García-Hermoso, María Correa-Rodríguez, Joaquín Fernández-Irigoyen, Sara Palomino-Echeverría, Enrique Santamaría, Jorge Enrique Correa-Bautista, Katherine González-Ruíz, Mikel Izquierdo

**Affiliations:** 1 Navarrabiomed, Hospital Universitario de Navarra (HUN), Navarra Institute for Health Research (IdiSNA), Universidad Pública de Navarra (UPNA), 31008 Pamplona, Spain; 2 Centro de Investigación Biomédica en Red de Fragilidad y Envejecimiento Saludable (CIBERFES), Instituto de Salud Carlos III, 28029 Madrid, Spain; 3 Escuela de Ciencias de la Actividad Física, el Deporte y la Salud, Facultad de Ciencias Médicas, Universidad de Santiago de Chile, USACH, Santiago 9170022, Chile; 4 Department of Nursing, Faculty of Health Sciences, University of Granada, 18016 Granada, Spain; 5 Biosanitary Research Institute (ibs.GRANADA), Granada, Spain; 6 Proteored-Institute of Health Carlos III (ISCIII), Clinical Neuroproteomics Unit, Navarrabiomed, Navarra Health Department, Public University of Navarra, Navarra Institute for Health Research (IdiSNA), 31008 Pamplona, Spain; 7 Translational Bioinformatics Unit (TransBio), Navarrabiomed, Navarra Health Department, Public University of Navarra, Navarra Institute for Health Research (IdiSNA), 31008 Pamplona, Spain; 8 Grupo Rendimiento Físico Militar “RENFIMIL”, Escuela Militar de Cadetes “General José María Córdova”, Bogotá, 111711, Colombia; 9 Grupo de Investigación Salud y Movimiento. Programa de Fisioterapia. Facultad de Salud, Universidad Santiago de Cali, 760035, Colombia; 10 Programa de Doctorado en Ciencias Biomédicas y Biológicas, Escuela de Medicina y Ciencias de la Salud - Facultad de Ciencias Naturales y Matemáticas, Universidad del Rosario, Bogotá, 111221, Colombia

**Keywords:** exercise, inflammation, youth, obesity

## Abstract

**Propose:**

Obesity-related metabolic risk factors in adolescents who are overweight/obese may be associated with systemic low-grade inflammation; therefore, we investigated whether 6 months of exercise training altered markers of inflammation.

**Methods:**

Secondary analyses of a randomized controlled exercise-based intervention trial (September 2017-December 2018). Adolescents aged 11 to 17 years (Tanner stage II-V), 70% girls, with a body mass index *z*-score at or above the 85th percentile, and/or with excess of adiposity (body fat ≥ 30%). The participants were randomly assigned to the following 4 groups for 6 months: (1) standard physical education lessons, as a control (CTRL); (2) high-intensity physical education class (HIPE); (3) low-to-moderate intensity physical education class (LIPE); (4) a combined group (PLUS). Inflammatory markers and immune molecules including chemokines, cytokines, and growth factors (n = 65 biomarkers) were determined by cytokine antibody array.

**Results:**

Of the 120 randomly assigned participants, 95 were included in the analysis. Considering these 22 proteins, the LIPE group shows statistical significance in 9 proteins with log-fold change (logFC) and *P* < 0.05 (in BLC, eotaxin, fibroblast growth factor-6 [FGF-6], GCP-2, I-309, IGFBP-4, MCP-4, NAP-2, and PARC), followed by the PLUS group in 9 proteins (BLC, pro-epidermal growth factor, eotaxin, FGF-6, MCP-4, NAP-2, osteopontin, PARC, and RANTES), the HIPE group in 7 proteins (FGF-4, FGF-7, GCP-2, IGF-1, IGFBP-1, IGFBP-4, and MIP-1 delta), and the CTRL group in 6 proteins (FGF-4, IP-10, Leptin, MCP-1, MIG, and MIP-1 delta). However, subanalysis performed to detect differentially expressed proteins at baseline and after intervention, with significance at an adjusted *P* value ≤ 0.05 and absolute log fold-change (logFC) ≥ 1.0, showed 3 downregulated proteins in the LIPE group (BLC_(logFC)_ = 1.27, eotaxin_(logFC)_ = 1.18, and MCP-4_(logFC)_ = 1.14), and 4 proteins in the HIPE group (BLC_(logFC)_ = 1.45, FGF-6_(logFC)_ = 1.20, MCP-4_(logFC)_ = 1.50, and PARC_(logFC)_ = 1.33), supporting that the changes we observed in the exercise groups were not time-related changes but occurred in response to exercise.

**Conclusions:**

Implementing a 6-month physical exercise program in overweight/obese adolescents, based on LIPE and PLUS groups, significantly change several circulating inflammatory levels. Interventions involving supervised physical exercise may reduce the associated effects of systemic low-grade inflammation, thus preventing the development of obesity-related metabolic diseases in adolescents with overweight/obesity.

The epidemic rate of obesity in adolescents has recently been recognized worldwide as a critical health issue. This is particularly important in South American adolescents, who have high rates of obesity (1). In addition, a lack of physical activity, along with other factors such as poor diet and a sedentary lifestyle, have been associated with the initiation and progression of obesity and obesity-associated metabolic comorbidities among adolescents (2). For this reason, both diet modification (3) and physical exercise/activity interventions (4) are considered the first lines of approach for treating obese children and adolescents. In this context, we reported that increasing supervised exercise in overweight and obese adolescents, independent of caloric restriction, is beneficial for reducing hepatic lipid composition, blood lipid profile, visceral and subcutaneous adipose tissue (5), and improving cardiorespiratory fitness (6) and inflammatory state (7).

The translation of physical activity to health effects rests on the interaction of seemingly dichotomous anabolic and catabolic mediators and cell signaling pathways, including growth factors growth factors and immune and inflammatory mediators including peripheral blood mononuclear cells and circulating pro- and anti-inflammatory cytokine (6). There exist critical periods of growth and development during which the effect of exercise on growth mediators and stress/inflammatory factors would have long-term health effects (8). Such periods occur early in life (particularly in premature babies) and in the pubertal transition.

However, the clinician attempting to prescribe a program of exercise training for children and adolescents with overweight/obesity faces a dilemma. Additionally, the utility of school-based exercise programs (with no caloric restriction) as a strategy for reducing obesity-related metabolic risk factors is unclear (9). A previous meta-analysis suggested a positive effect of school-based interventions on physical fitness (10, 11); however, the optimal exercise regimen for reducing adiposity and its cardiometabolic risk factors such as inflammatory biomarkers have not been fully clarified. In the past few years, several studies have demonstrated that nontraditional biomarkers that function as regulators of metabolic function may be involved in the etiopathogenesis of nonalcoholic fatty liver disease and other metabolic disorders (12-14). As far as we are aware, there are no previous studies identifying the profile of nontraditional biomarkers in adolescents at a high risk of metabolic disorders and the response to interventions involving exercise.

Recently, we reported that 6-month physical education exercise program, particularly high intensity or combined high and low intensity, improves hepatic fat storage and significantly reduces cardiometabolic markers in adolescents with excess of adiposity (15). A unique feature of the study was that all completed exercise was supervised throughout the entire intervention. However, the effect of varying exercise dose on the inflammatory marker levels were not explored. Another knowledge gap concerns questions regarding the physiological mechanisms that underlie the beneficial effects of exercise induced to reduce metabolic disorders associated with obesity progression, such as nonalcoholic fatty liver disease, inflammation, and insulin resistance. No study has yet identified the physiological mechanisms that mediate the beneficial effects of aerobic exercise among adolescents with overweight/obesity.

Therefore, we conducted a randomized controlled trial to examine the role of regular exercise on its own (ie, no calorie restriction) on markers of inflammation in adolescents with overweight/obesity. We hypothesized that 6-month exercise program would produce effects on these inflammation markers independently of dose protocol, which would underscore the notion that exercise benefits among adolescents with overweight/obesity from Bogotá, Colombia.

## Methods/Design

### Study Design

This study was reviewed and approved by research ethics boards of the Medical Research Ethics Committee of The University of Rosario (Code UR-CEI-ABN026-000140) and complied with the revised ethical guidelines of the Declaration of Helsinki (2013 revision). Description and characteristics of the study were given to parents or legal guardians, and a written informed consent was provided from both guardian and adolescent. Full details of the original trial protocol are published (15, 16), and participants were recruited from a single official school (in Spanish, Colegio Instituto Técnico Internacional) from Bogotá, Colombia.

### Participants

Participants (Table 1), who were Tanner stage II to V adolescents aged 11 to 17 years (70% girls) with overweight/obese status by body mass index (BMI) *z*-score according to the International Obesity Task Force (17) and/or excess of adiposity (body fat ≥ 30%), were enrolled in the program sessions. Exclusion criteria included habitual exercise more than twice weekly for more than 60 minutes per session, pregnancy, cardiovascular disease, diabetes mellitus (type 1 or 2), or any illness or disability rendering study physical education lessons inadvisable or unfeasible. Additionally, participants with other causes of liver disease featuring elevated liver enzymes and/or hepatotropic viruses (ie, positive for hepatitis B surface antigen, anti-hepatitis Bc/anti-hepatitis C virus/anti-HIV antibodies, and/or others) were excluded. Routine school physical education classes were not an exclusion criterion. Details about randomization and allocation procedures and sample size considerations have been published elsewhere (16).

**Table 1.** Baseline characteristics of participants

Characteristics	CTRL group (n = 26)		HIPE group (n = 21)		LIPE group (n = 25)		PLUS group (n = 23)		All participants (n = 95)	
Girls/boys, n^*a*^	18/8		12/9		19/6		18/5		67/28	
Chronological age, y	13.9	(1.6)	13.9	(1.4)	13.4	(1.7)	12.8	(1.8)	13.5	(1.6)
BMI, *z*-score	1.8	(0.4)	1.8	(0.4)	1.9	(0.7)	1.8	(0.6)	1.8	(0.5)
Body fat,%	39.5	(4.7)	39.4	(3.9)	41.0	(4.9)	39.5	(4.9)	39.9	(4.6)

Abbreviations: BMI, body mass index; CTRL, control; HIPE, high-intensity physical education class; LIPE, low-to-moderate intensity physical education class; PLUS, combined HIPE/LIPE group.

^*a*^Results are shown as mean and SD for continuous variables and percentages for categorical variables.

### Intervention

Participants entered a run-in period that included school-based exercise programs, 3 times weekly for 6 months. Risks was minimized by identifying contraindications to the testing and training protocols via a health history and a thorough physician examination before the testing sessions. Details about interventions have been described (16). In terms of training intensity, the actual values were the mean heart rates measured in the high-intensity physical education class (HIPE), low-to-moderate intensity physical education class (LIPE), or a combined group (PLUS) groups in a subsample (25 participants). Heart rate monitors (Polar FS1; Polar Electro, Oy, Finland) were used to adjust workloads to achieve target heart rates. Compliance to the exercise protocol, an essential element of an efficacy study, was defined as successfully completing > 70% of scheduled exercise sessions, maintaining the target exercise heart rate ± 4 beats/min^-1^ for the prescribed duration of the playground games session. Details of the exercise programs have been published elsewhere (16). All study participants received a hygiene–dietary management plan that included educational and nutritional support throughout the study.

### Inflammatory Markers

In this extension study, venous blood samples were obtained from an antecubital vein around 7:00 to 9:00 am following a 10- to 12-hour overnight fast. Samples were handled according to the Clinical Laboratory Improvement Amendments, which must be followed to achieve valid test results that can be used for diagnoses. Blood was centrifuged at 1000*g* for 10 minutes at 4°C and stored at -80°C for future analysis. Quantification of 80 analytes in serum samples was performed using the Abcam Human Cytokine Antibody Array (80 targets; #ab133998) (Myriad RBM, Austin, TX). Briefly, dot-blot protein arrays with 80 cytokine antibodies were blocked with the manufacturer’s blocking buffer at room temperature for 30 minutes, and then incubated overnight with 200 to 250 μg of serum from all patients. Blocking buffer was aspirated and undiluted cell culture supernatants (1 mL) were added to each well and incubated overnight at 4°C. After sample incubation, membranes were thoroughly washed and incubated with 1 mL of 1X Biotin-Conjugated Anti-Cytokines overnight at 4°C. Then the membranes were washed again and incubated with 2 mL of 1X HRP-Conjugated Streptavidin for 2 hours at room temperature. The membranes were then washed, and the cytokines expressed were detected by chemiluminescence with the ImageQuant ECL system (BioRad, Madrid, Spain) and densitometric data obtained using ImageJ software. Mean intensities of negative and positive control spots were used for background correction and normalization, respectively. The array detects the following cytokines: ENA-78 (CXCL5), granulocyte colony-stimulating factor, granulocyte-macrophage colony-stimulating factor, GRO (CXCL1), GRO-alpha (CXCL2), I-309 (CCL1), IL-1alpha, IL-1beta, IL-2, IL-3, IL-4, IL-5, IL-6, IL-7, IL-8 (CXCL8), IL-10, IL-12, IL-13, IL-15, interferon-gamma, MCP-1 (CCL2), MCP-2 (CCL8), MCP-3 (CC7), macrophage colony-stimulating factor, MDC (CCL22), MIG (CXCL9), MIP-1beta (CCL4), MIP-1delta (CCL15), RANTES (CCL5), mast cell growth factor or C-kit ligand, SDF-1 (CXCL12), TARC (CCL17), TGF-beta1, TNF-alpha, TNF-beta, pro-epidermal growth factor, IGF-I, angiogenin, oncostatin M, thrombopoietin, vascular endothelial growth factor, platelet-derived growth factor subunit B, leptin, brain-derived neurotrophic factor, BLC (CXCL13), Ckß8-1 (MIP-3/CCL23), eotaxin (CCL11), eotaxin-2 (CCL24), eotaxin-3 (CCL26), fibroblast growth factor-4 (FGF-4), FGF-6, FGF-7, FGF-9, Flt-3 Ligand, fractalkine, GCP-2 (CXCL6), glial cell line-derived neurotrophic factor, hepatocyte growth factor, IGFBP-1, IGFBP-2, IGFBP-3, IGFBP-4, IL-16, IP-10 (CXCL10), leukemia inhibitory factor, TNF ligand superfamily member 14, MCP-4 (CCL13), macrophage migration inhibitory factor, MIP-3 alpha (CCL20), NAP-2 (CXCL7), neurotrophin-3, neurotrophin-4, osteopontin, osteoprotegerin, PARC (CCL18), placenta growth factor), TGF-beta2, TGF-beta3, metalloproteinase inhibitor-1, and metalloproteinase inhibitor-2.

### Statistical Analysis

The original analysis plan specified a repeated-measures mixed model; however, because the model did not converge, we used the simpler model without repeated measures. Cytokine antibody array expression data were measured as intensities, which needed to be background corrected and normalized before statistical analysis. Preprocessing of cytokine expression levels was applied as described by González-Morales et al (18). In addition, the log_2_ transformed data were normalized using the quantile algorithm. Only cytokines with fewer than 5% missing values were considered for further analysis. Values are presented in arbitrary units. The *limma* R package was used to compute the statistical significance among experimental groups and time (19). The significance level was adjusted considering a false discovery rate (FDR) of 0.1 for the purpose of corrections because of multiple tests. Last, Search Tool for the Retrieval of Interacting Genes software (v.11) (http://stringdb.org/) was used to extract functional relationships between cytokines and growth factors in each experimental group (20). Statistical analyses were performed using SPSS, version 25 (IBM Corp, Chicago, IL) software, SAS, version 9.2 (SAS Institute, Cary, NC) commercially available software, and R software (version 3.6.2).

## Results

**Figure 1.**
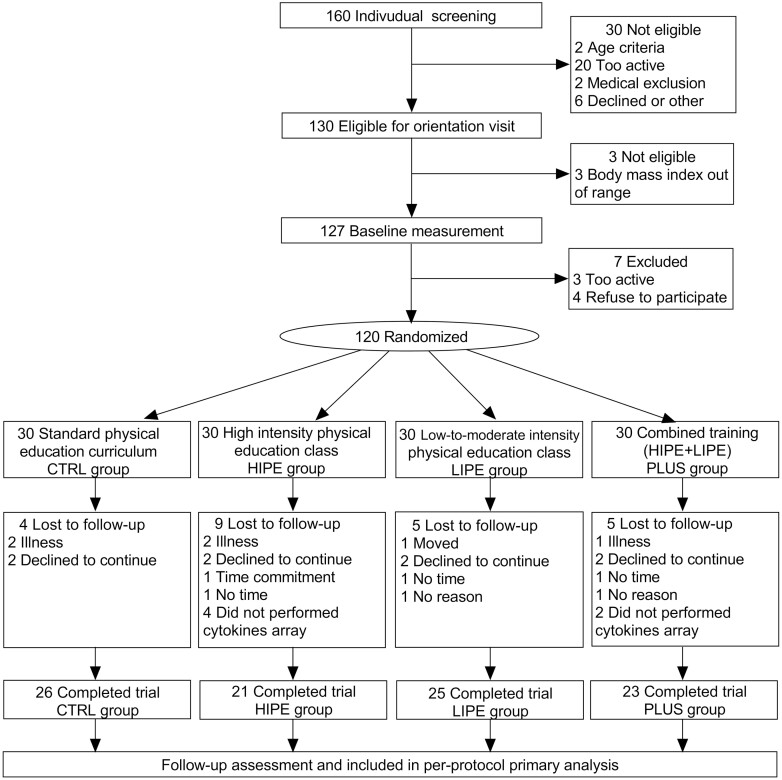
Participant flow diagram.

A total of 160 adolescents were screened. According to the exclusion and inclusion criteria, 33 potential subjects (20.6%) were ineligible. After giving informed consent, 127 (79%) were submitted to baseline medical and clinical measurements: 120 (75%) had complete full data at baseline and were randomized; and 95 (79%) participants completed the study and had data on all variables used in the present secondary analyses. The incidence of unrelated adverse events occurred in 2 of 21 HIPE group participants (10%), in 1 of 23 PLUS group participants (4%), and in 1 of 23 in the control group (CTRL) (4%). Almost all related adverse events involved musculoskeletal injury or discomfort and upper/lower body injury (minor injury or falls) such as bruises (contusions). No medical adverse events were related to the intervention. Reasons for withdrawal are shown in Fig. 1.

The descriptive characteristics for the study sample are presented in Table 1. Baseline characteristics were similar among participants assigned to the 4 interventions and between those who were assigned to a interventions and those who completed the study. Participant characteristics were generally in accord with those observed in population studies with predominantly girls (n = 67, 70%). The mean age of the participants was 13.5 (1.6) years, the mean BMI *z*-score was 1.8 (0.5), and the mean body fat was 39.9 (4.6)%. The information regarding the hepatic fat content (primary endpoint), secondary outcomes such as traditional cardiovascular health markers (body composition, serum lipids, aminotransferases and health-related physical fitness components), and diet intake were described in a previous publication (16).

Change in body fat (%) in response to exercise is presented in Supplemental Figure S1 (21). A body fat decreased in the HIPE group -2.88% (95% CI, -3.96 to -1.81, *P* = 0.001) and the LIPE group -0.62% (95% CI, -1.68 to -0.45, *P* = 0.014), independent for dietary intake (Supplemental Figure S2) (22), suggesting that 6 months of moderate- to high-intensity exercise and/or combined protocol were effective in reducing these biomarkers of inflammation.

**Figure 2.**
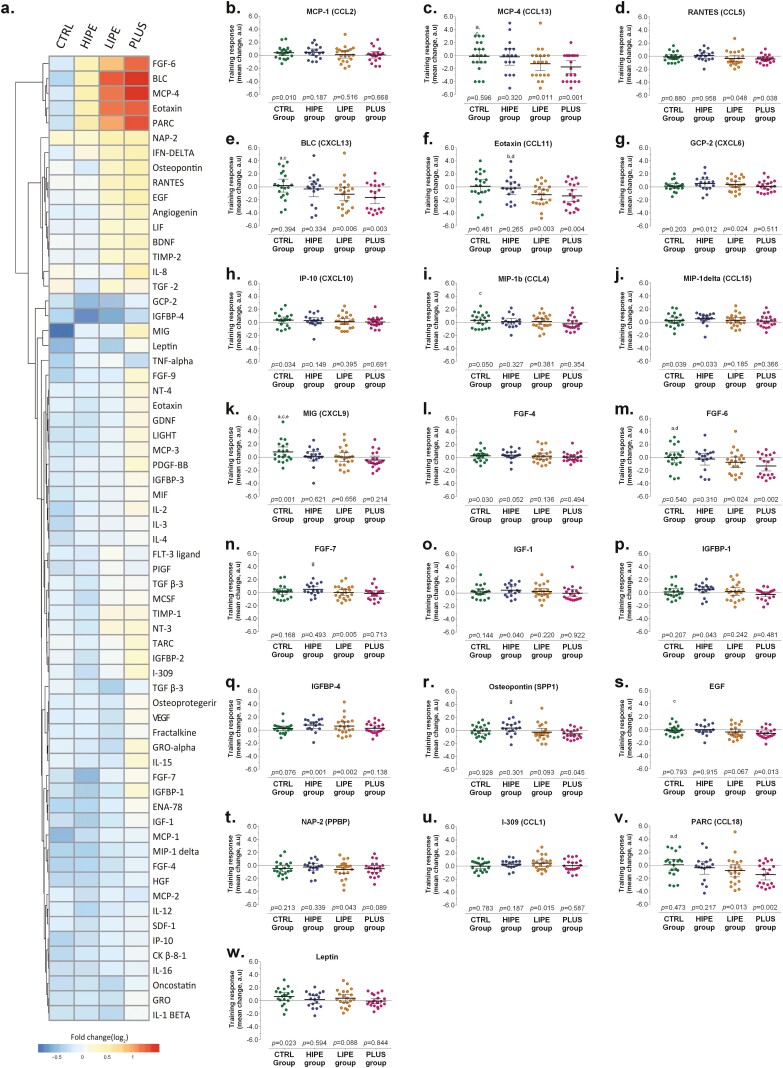
Change in inflammatory markers and immune molecules including chemokines, cytokines, and growth factors in response to exercise (log fold-change) from the baseline in 22/65 proteins. The letters (a-g) represent comparison of least squares means of the outcome. (a) CTRL vs LIPE *P* < 0.05; (b) CTRL vs LIPE *P* < 0.01; (c) CTRL vs PLUS *P* < 0.05; (d) CTRL vs PLUS *P* < 0.01; (e) CTRL vs HIPE *P* < 0.05; (f) CTRL vs HIPE *P* < 0.01; (g) HIPE vs PLUS *P* < 0.05.

The array proteins were selected given their relevance to obesity, insulin resistance, and hepatic steatosis, and they consisted of inflammatory markers and immune molecules including chemokines, cytokines, and growth factors. Figure 2 shows that the effects of the interventions on pattern of change in response to exercise (log fold-change [_logFC_]) differed from the baseline in 22/65 proteins (median = 33.8%). Considering these 22 proteins, the LIPE group shows statistical significance in 9 proteins _(logFC)_ > 0.3 and *P* < 0.05, was found in BLC, eotaxin, FGF-6, GCP-2, I-309, IGFBP-4, MCP-4, NAP-2, and PARC, followed by the PLUS group in 9 proteins (BLC, pro-epidermal growth factor, eotaxin, FGF-6, MCP-4, NAP-2, osteopontin, PARC, and RANTES), the HIPE group in 7 proteins (FGF-4, FGF-7, GCP-2, IGF-1, IGFBP-1, IGFBP-4, and MIP-1 delta), and the CTRL group in 6 proteins (FGF-4, IP-10, Leptin, MCP-1, MIG, MIP-1 delta) (Fig. 2A). However, subanalysis performed to detect differentially expressed proteins at baseline and after intervention, with significance at an adjusted *P* value ≤ 0.05 and absolute (logFC) ≥ 1.0, showed 3 downregulated proteins in the LIPE group (BLC_(logFC)_ = 1.27, eotaxin_(logFC)_ = 1.18, and MCP-4_(logFC)_ = 1.14), and 4 proteins in the HIPE group (BLC_(logFC)_ = 1.45, FGF-6_(logFC)_ = 1.20, MCP-4_(logFC)_ = 1.50, and PARC_(logFC)_ = 1.33), supporting that the changes that we observed in the exercise groups were not time-related changes but occurred in response to exercise (Fig. 2B-W).

A significant time × group interaction (logFC > 1.0) was found in the LIPE group in BCL_(logFC)_ = 1.65, eotaxin_(logFC)_ = 1.45, FGF-6_(logFC)_ = 1.04, and PARC_(logFC)_ = 1.27 (*P* < 0.05 vs CTRL group). Also, BLC_(logFC)_ = 1.83, eotaxin_(logFC)_ = 1.50, FGF-6_(logFC)_ = 1.42, MCP-4_(logFC)_ = 1.73, MIG_(logFC)_ = 1.26, and PARC_(logFC)_ = 1.60 decreased in the PLUS group (*P* < 0.05 vs CTRL group). Five of the 65 interleukins/chemokines (MIP-1b, RANTES, BCL, eotaxin, and PARC), in addition to FGF-6, decreased significantly in response to exercise among the adolescents from the HIPE and PLUS groups (*P* < 0.05).

**Figure 3.**
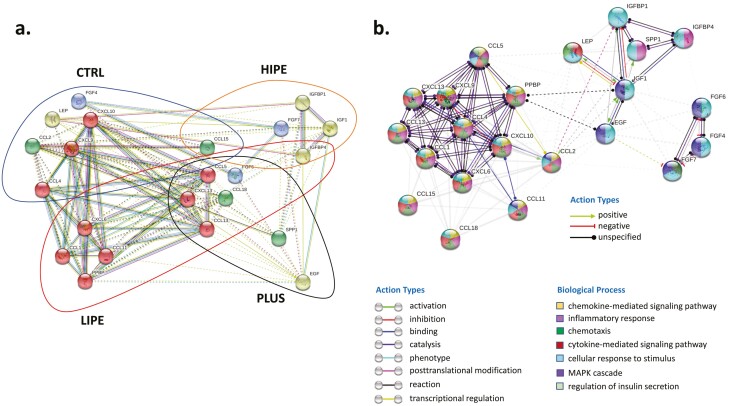
Interactome network for deregulated cytokines. (A) Network analysis was performed submitting the corresponding protein identifications to the STRING (Search Tool for the Retrieval of Interacting Genes) software (v.11) (http://stringdb.org/). Only interactions tagged with > 0.4 confidence in the STRING database were considered. (B) Functional protein interactome networks were constructed, revealing commonalities and differences in the known functional relationships established between deregulated cytokines and growth factors observed at follow-up. The proteins are represented with nodes and edges (physical or functional interactions), supported by at least 2 reference from the published literature or from canonical information stored in the STRING database.

As shown in Fig. 3, the monitoring of cytokine expression in all the groups reveals certain overlap. IGFBP-4 and CXCL6 were commonly deregulated in the LIPE and HIPE groups, whereas the levels of BLC (CXCL13), eotaxin (CCL11), FGF-6, MCP-4 (CCL13), PARC (CCL18), and RANTES (CCL5) decreased in the LIPE and PLUS groups. Functional protein interactome networks were constructed, revealing commonalities and differences in the known functional relationships established between deregulated cytokines and growth factors observed at follow-up (Fig. 3A). As a whole, the fluctuations observed in the cytokine panel (Fig. 3B) suggest that the response to exercise generally affects important biofunctions such as chemokine-mediated signaling pathway (FDR: 4.31e-23), inflammatory response (FDR: 2.91e-17), chemotaxis (FDR: 1.56e-15), and cellular response to stimulus (FDR: 3.16e-11), among others (Supplemental Table S1) (23).

## Discussion

The primary finding from this randomized, controlled exercise trial involving adolescents with overweight/obesity is that both LIPE and PLUS groups were associated with significant reductions in the differential expression of several cytokine levels after 6 months of 4 different physical training regimens (HIPE, LIPE, PLUS, and CTRL), supporting the anti-inflammatory effect of these training programs. Our results are in line with the international recommendations of physical activity (2018 Physical Activity Guidelines recommendation) that physical activity programs consist of moderate- to vigorous-intensity aerobic physical activity combined with resistance training in children/adolescents aged 6 to 17 years (24), which recommend 60 minutes of aerobic and muscle strengthening, supervised exercise training at moderate and vigorous intensity every day or at least 3 days per week in this population (25).

In our study we found that serum levels of MIP-1b (CCL4), RANTES (CCL5), BLC (CXCL13), eotaxin (CCL11), PARC (CCL19), and FGF-6 decreased significantly in the LIPE and PLUS groups, supporting the positive effect of the training programs and providing novel insights into the mechanisms underlying the role of high-intensity physical activity and aerobic training programs plus resistance training. MIP-1b (CCL4) is a proinflammatory chemokine that promotes leukocyte accumulation in various inflammatory conditions. Interestingly, previous research found that the expression of MIP-1b and RANTES increased as a consequence of weight gain in obese mice (26). It could therefore be hypothesized that the weight loss accompanying the physical training could be the reason for the decrease in these chemokines. Additionally, RANTES (CCL5) is a powerful pro-inflammatory mediator of the CC chemokine family that regulates mobilization and, in certain cases, promotes immune cell survival in areas of injury and infection (27). Despite its beneficial chemotactic activity on immunity in injured and infected areas, in obese subjects, its elevated levels are associated with atherosclerosis (28) and hepatic involvement (29). In our study, we observed a significant decrease in their levels among obese adolescents in the LIPE and PLUS intervention groups. Other studies, such as that by Baturcam et al (30), show that a 3-month training program combining moderate-intensity aerobic exercise and resistance training reduces the expression of RANTES and its receptor (CCR5) in the subcutaneous adipose tissue, although it has only a marginal effect on circulating RANTES levels. Although further studies are required, these findings seem to indicate that physical exercise may be one of the nonpharmacological approaches able to attenuate the RANTES signaling pathway, acting on its circulating levels and thus mitigating the inflammatory and metabolic stress triggered by obesity.

On the other hand, it has been established that the chemokine C-X-C motif ligand 13 (CXCL13)/BLC and its receptor, the G-protein coupled receptor CXCR5, play crucial roles in inflammatory, infectious, and immune responses (31). Furthermore, CXCL13 has been shown to be produced at elevated levels during adipogenesis, and CXCL13 was also highly expressed in white adipose tissue from mice fed a high-fat diet (32). However, its potential role in hepatic fat and cardiovascular health has not previously been investigated. Similarly, there are no previous studies analyzing the effect of supervised physical exercise on interleukin eotaxin (CCL11) levels in obese adolescents; all studies having been carried out in adult populations (33). In our study, the reduced levels observed in the adolescents in the LIPE and PLUS intervention groups could be explained by a possible modulating effect of moderate-intensity aerobic exercise and resistance training on circulating levels of this interleukin.

We also found decreased expression of PARC (CCL18), a chemokine produced by antigen-presenting cells and macrophages in adipose tissue, in the LIPE and PLUS groups. Although no similar studies have been conducted, CCL18 levels have been shown to be higher in obese patients and positively correlated with an Adult Treatment Panel III risk score, triglycerides, TNF-α, and IL-6 (34). Our data showed that serum levels of FGF6, a member of the FGF family accumulating almost exclusively in the myogenic lineage (35), decreased in the obese adolescents enrolled in the LIPE and PLUS groups. In line with the results of Pedersen (36) on the anti-inflammatory properties of physical exercise, our results suggest the utility of high-intensity aerobic and resistance training as a means of modulating the levels of certain pro-inflammatory interleukins in adolescent subjects, thus performing an important role in protecting against diseases associated with low-grade inflammation, such as cardiovascular disease and type 2 diabetes. To our knowledge, because this is the first study to examine the differential expression of these cytokine levels after 6 months of different physical training regimens, our preliminary findings should be analyzed further in future randomized clinical trials involving adolescents in a school setting.

In addition, we observed a significant reduction in circulating levels of pro-inflammatory interleukins such as MCP-4 (CCL13) in those overweight/obese adolescents who received physical education at various intensities (LIPE, HIPE, or PLUS groups). CCL13 is induced by inflammatory proteins such as IL-1 and TNF-α. MCP*-*4 activates signaling in monocytes, T lymphocytes, eosinophils, and basophils, and this signaling is associated with the recruitment of monocytes into the arterial wall during atherosclerosis. Furthermore, MCP-4 is a novel biomarker of severe obesity and could play an indirect role in favoring subclinical atherosclerosis in obese patients by influencing the circulating levels of eotaxin-3 and MIP-1b, which are directly related to the main atherosclerosis markers (37). Previously, a study showed that MCP-4 is a critical molecule that links obesity with chronic inflammation, serum levels of MCP-4 correlate with BMI, and it has been identified as a potential therapeutic target (38). To our knowledge, this study is the first to indicate that exercise may decrease MCP-4 plasma levels in vivo. The more extensive lowering of MCP-4 in the LIPE and PLUS groups (Fig. 2C) suggests an additive, suppressive effect of physical education and fitness on MCP-4 levels in plasma. In light of our findings, it is therefore possible that the beneficial effect of exercise with regard to cardiovascular health could be due, at least in part, to reduced MCP-4.

Interestingly, we found that LIPE (low-to-moderate intensity physical education class) induces an adaptive immune response to attenuate proinflammatory cytokine expression and benefit the host. We hypothesize that the increases in light physical activity intensity mimic regular training exercise and therefore could improve subclinical inflammation in adolescents in the overweight/obese population. These results support the new global physical activity guidelines recommendation of physical activity programs consisting of light to moderate-vigorous intensity aerobic physical activity combined with resistance training in children/adolescents aged 6 to 17 years (39). However, the potential effects of supervised physical exercise on innate immune function in adolescents who are overweight/obese still need further investigation.

Several studies reported a close relationship between exercise and concentration of circulating cytokines such as IL-1β, TNF-α, IL-6, IL-10, and INF-γ in response to acute and/or chronic exposure to exercise (40). Results of our study in cases of unseen significant decreases in IL-1, IL-6, and TNF-α, especially in supervised exercise groups, are the same as in other foundlings (40-42). For example, Kim et al (43). reported no significant changes in IL-1, IL-6, and TNF-α after endurance training was observed in obese male Korean adolescents. In a recent systematic review, based on 115 overweight and obese youths, Han et al (44) suggest that exercise training does not significantly mitigate IL-6 levels (mean difference =  – 0.39 pg/mL, *P* = 0.08), although there was a trend toward a reduction. Additionally, no close connection was observed between exercise and TNF-α levels at 0.04 pg/mL (*P* = 0.78) in overweight or obese children or adolescents. On the contrary, Alizadeh et al (45) reported that 6 weeks of high-intensity, intermittent training sessions 3 days/wk for 6 weeks significantly increased the baseline serum levels of IL-4 (*P* = 0.022) and IL-13 (*P* = 0.014) in overweight adolescent boys. These disparities between studies may be explained in part by differences in the exercise protocols adopted. Thus, evidence of the effect of exercise alone on controlling inflammatory cytokine (pro- or anti-inflammatory) levels in overweight or obese children remains limited. For this reason, subsequent studies must delineate the mechanisms through which the inflammatory process is affected by exercise without dietary restriction.

Compared with previous studies, our study had a relatively modest sample size and was conducted in the school setting, where most adolescents with overweight/obesity are found. Nevertheless, the study has several limitations. First, the research was conducted on a population of adolescents with excess adiposity; the findings may not be generalizable to thin subjects. Second, the nature of intervention made blinding of adolescents impossible. Unmeasured changes other than dietary (eg, 24-hour recall) and physical activity pattern might have contributed to alleviating chronic inflammation through body fat reduction. Dietary composition is probably less important than total energy intake, although a low-carbohydrate diet may be slightly more effective than the alternatives if it is acceptable to the individual (46). However, during interventions involving exercise, fat mass will be lost and muscle mass will be gained, and these opposing changes can result in lower production of pro-inflammatory cytokines from peripheral mononuclear cells in individuals with an elevated risk for cardiovascular disease, including adolescents who are overweight/obese.

The main strength of our study is that it is the first to compare the differential expression of several cytokine levels measured using an antibody-based membrane array after 6 months of different physical activity regimens (HIPE, LIPE, PLUS, and CTRL). Additionally, highly standardized procedures have been developed within the Exercise Training and Hepatic Metabolism in Overweight/Obese Adolescents study to avoid measurement bias.

In conclusion, implementing a 6-month physical exercise program for overweight/obese adolescents based on LIPE and PLUS changes a number of circulating interleukin levels, some of which are pro-inflammatory (eg, MIP-1b/CCL4, BCL/CXC13), thus decreasing systemic inflammation in these subjects. In addition, and because adolescence is a critical period with respect to adopting health maintenance behaviors, it is necessary to promote strategies that strengthen healthy habits during these stages. In this sense, it is postulated that interventions involving supervised physical exercise aimed at preventing metabolic dysfunction could help improve the effects of systemic low-grade inflammation, thus preventing the development of obesity-related metabolic diseases in adolescents who are overweight/obese.

## Data Availability

The original contributions presented in the study are included in the article/supplementary material. Further inquiries can be directed to the corresponding author.

## Abbreviations

BMI: body mass index
CTRL: control
FDR: false discovery rate
FGF: fibroblast growth factor
HIPE: high-intensity physical education class
LIPE: low-to-moderate intensity physical education class
logFC: log fold-change
PLUS: combined high-intensity physical education class and low-to-moderate intensity physical education class

## Study Registration

ClinicalTrials.gov registration no. NCT02753231 (registered 27 April 2016).

## References

[1] NCD Risk Factor Collaboration (NCD-RisC) NRFC. Worldwide trends in body-mass index, underweight, overweight, and obesity from 1975 to 2016: a pooled analysis of 2416 population-based measurement studies in 128·9 million children, adolescents, and adults. Lancet. 2017;390(10113):2627-2642. 29029897 10.1016/S0140-6736(17)32129-3 PMC5735219

[2] Eker HH , Taşdemir M, Mercan S, et al Obesity in adolescents and the risk factors. Turk J Phys Med Rehabil. 2018;64(1):37-45. 31453487 10.5606/tftrd.2018.1402 PMC6709607

[3] Andela S , Burrows TL, Baur LA, Coyle DH, Collins CE, Gow ML. Efficacy of very low-energy diet programs for weight loss: a systematic review with meta-analysis of intervention studies in children and adolescents with obesity. Obes Rev. 2019;20(6):871-882. 30734459 10.1111/obr.12830

[4] García-Hermoso A , Ramírez-Vélez R, Saavedra JM. Exercise, health outcomes, and pædiatric obesity: a systematic review of meta-analyses. J Sci Med Sport. 2019;22(1):76-84. 30054135 10.1016/j.jsams.2018.07.006

[5] González-Ruiz K , Ramírez-Vélez R, Correa-Bautista JE, Peterson MD, García-Hermoso A. The effects of exercise on abdominal fat and liver enzymes in pediatric obesity: a systematic review and meta-analysis. Child Obes. 2017;13(4):272-282. 28322576 10.1089/chi.2017.0027

[6] Radom-Aizik S . Immune response to exercise during growth. Pediatr Exerc Sci. 2017;29(1):49-52. 28271813 10.1123/pes.2017-0003

[7] García-Hermoso A , Ramírez-Vélez R, Ramírez-Campillo R, Peterson MD, Martínez-Vizcaíno V. Concurrent aerobic plus resistance exercise versus aerobic exercise alone to improve health outcomes in paediatric obesity: a systematic review and meta-Analysis. Br J Sports Med. 2018;52(3):161-166. 27986760 10.1136/bjsports-2016-096605

[8] Cooper DM , Nemet D, Galassetti P. Exercise, stress, and inflammation in the growing child: from the bench to the playground. Curr Opin Pediatr. 2004;16(3):286-92. 15167015 10.1097/01.mop.0000126601.29787.39

[9] Pozuelo-Carrascosa DP , García-Hermoso A, Álvarez-Bueno C, Sánchez-López M, Martinez-Vizcaino V. Effectiveness of school-based physical activity programmes on cardiorespiratory fitness in children: a meta-analysis of randomised controlled trials. Br J Sports Med. 2018;52(19):1234-1240. 29074475 10.1136/bjsports-2017-097600

[10] Kriemler S , Meyer U, Martin E, Van Sluijs EMF, Andersen LB, Martin BW. Effect of school-based interventions on physical activity and fitness in children and adolescents: a review of reviews and systematic update. Br J Sports Med. 2011;45(11):923-30. 21836176 10.1136/bjsports-2011-090186 PMC3841814

[11] Dobbins M , DeCorby K, Robeson P, Husson H, Tirilis D. School-based physical activity programs for promoting physical activity and fitness in children and adolescents aged 6–18. Cochrane Database Syst Rev. 2009(1). doi 10.1002/14651858.CD007651. 19160341

[12] Goldner D , Lavine JE. Nonalcoholic fatty liver disease in children: unique considerations and challenges. Gastroenterology. 2020;158(7):1967-1983.e1. 32201176 10.1053/j.gastro.2020.01.048

[13] Lavine JE , Vittorio J. Recent advances in understanding and managing pediatric nonalcoholic fatty liver disease. F1000Research. 2020;9. doi:10.12688/F1000RESEARCH.24198.1/DOI. PMC7238455 32509277

[14] Murphy ECS , Carson L, Neal W, Baylis C, Donley D, Yeater R. Effects of an exercise intervention using Dance Dance Revolution on endothelial function and other risk factors in overweight children. Int J Pediatr Obes. 2009;4(4):205-14. 19922034 10.3109/17477160902846187

[15] González-Ruíz K , Correa-Bautista JE, Izquierdo M, et al Effects of an exercise program on hepatic metabolism, hepatic fat, and cardiovascular health in overweight/obese adolescents from Bogota, Colombia (the HEPAFIT study): study protocol for a randomized controlled trial. Trials. 2018;19(1). doi:10.1111/IJPO.12869. PMC6019229 29941024

[16] González-Ruíz K , Correa-Bautista JE, Izquierdo M, et al Exercise dose on hepatic fat and cardiovascular health in adolescents with excess of adiposity. Pediatr Obes. 2021. doi:10.1186/s13063-018-2721-5. 34734674

[17] Cole TJ , Bellizzi MC, Flegal KM, Dietz WH. Establishing a standard definition for child overweight and obesity worldwide: international survey. BMJ. 2000;320(7244):1240-1243. 10.1136/bmj.320.7244.1240 PMC27365 10797032

[18] González-Morales A , Lachén-Montes M, Fernandez-Irigoyen J, et al. Monitoring the cerebrospinal fluid cytokine profile using membrane-based antibody arrays. *Methods Mol Biol*. 2019;2044:233–246. 10.1007/978-1-4939-9706-0_14 31432416

[19] Ritchie ME , Phipson B, Wu D, et al limma powers differential expression analyses for RNA-sequencing and microarray studies. *Nucleic Acids Res.* 2015;43(7):e47-e47. 25605792 10.1093/nar/gkv007 PMC4402510

[20] Szklarczyk D , Gable AL, Lyon D, et al STRING v11: protein-protein association networks with increased coverage, supporting functional discovery in genome-wide experimental datasets. Nucleic Acids Res. 2019;47(D1):D607-D613. 30476243 10.1093/nar/gky1131 PMC6323986

[21] Ramírez-Vélez R , García-Hermoso A, Correa-Rodríguez M, et al. Change in body fat (%) in response to exercise by groups. Accessed January 22, 2022. Available at: https://figshare.com/articles/figure/Supplemental_Figure_S1/17091917

[22] Ramírez-Vélez R , García-Hermoso A, Correa-Rodríguez M, et al. Effects of different doses of exercise on quality of diet index (KIDMED score) among adolescents with overweight/obese. Accessed January 22, 2022. Available at: https://figshare.com/articles/figure/Suplemental_Figure_S1/17327309.

[23] Ramírez-Vélez R , García-Hermoso A, Correa-Rodríguez M, et al. STRING (Search Tool for the Retrieval of Interacting Genes) software (v.11) ( http://stringdb.org/ ) for extract functional relationships between cytokines and growth factors. Accessed January 22, 2022. Available at: https://figshare.com/articles/dataset/Table_S1_STRING_Search_Tool_for_the_Retrieval_of_Interacting_Genes_software_v_11_http_stringdb_org_for_extract_functional_relationships_between_cytokines_and_growth_factors_/17091953.

[24] World Health Organization. Global Action Plan on Physical Activity 2018–2030: More Active People for a Healthier World. Geneva: World Health Organization; 2018.

[25] Osiński W , Kantanista A. Physical activity in the therapy of overweight and obesity in children and adolescents. Needs and recommendations for intervention programs. Dev Period Med. 2017;21(3):224-234. doi: 10.34763/devperiodmed.20172103.224234. 29077562 PMC8522954

[26] Surmi BK , Webb CD, Ristau AC, Hasty AH. Absence of macrophage inflammatory protein-1α does not impact macrophage accumulation in adipose tissue of diet-induced obese mice. Am J Physiol Endocrinol Metab. 2010;299(3). doi: 10.1152/ajpendo.00050.2010. PMC2944285 20551286

[27] Keophiphath M , Rouault C, Divoux A, Clément K, Lacasa D. CCL5 promotes macrophage recruitment and survival in human adipose tissue. Arterioscler Thromb Vasc Biol. 2010;30(1):39-45. 19893003 10.1161/ATVBAHA.109.197442

[28] Ross R . Inflammation or atherogenesis. N Engl J Med. 1999;340(2):115-26. 9887164 10.1056/NEJM199901143400207

[29] Schwabe RF , Bataller R, Brenner DA. Human hepatic stellate cells express CCR5 and RANTES to induce proliferation and migration. Am J Physiol Gastrointest Liver Physiol. 2003;285(5):G949-58. doi:10.1152/ajpgi.00215.2003. 12829440

[30] Baturcam E , Abubaker J, Tiss A, et al Physical exercise reduces the expression of RANTES and its CCR5 receptor in the adipose tissue of obese humans. Mediat Inflamm. 2014:2014. doi:10.1155/2014/627150. PMC4016945 24895488

[31] Kazanietz MG , Durando M, Cooke M. CXCL13 and its receptor CXCR5 in cancer: inflammation, immune response, and beyond. Front. Endocrinol. 2019;10(JULY):471. 10.3389/fendo.2019.00471 PMC6639976 31354634

[32] Kusuyama J , Bandow K, Ohnishi T, et al CXCL13 is a differentiation- and hypoxia-induced adipocytokine that exacerbates the inflammatory phenotype of adipocytes through PHLPP1 induction. Biochem J. 2019;476(22):3533-3548. 31710352 10.1042/BCJ20190709

[33] Choi KM , Kim JH, Cho GJ, Baik SH, Park HS, Kim SM. Effect of exercise training on plasma visfatin and eotaxin levels. Eur J Endocrinol. 2007;157(4):437-42. 17893257 10.1530/EJE-07-0127

[34] Hogling DE , Petrus P, Gao H, et al Adipose and circulating CCL18 levels associate with metabolic risk factors in women. J Clin Endocrinol Metab. 2016;101(11):4021-4029. 27459538 10.1210/jc.2016-2390

[35] Armand AS , Pariset C, Laziz I, et al FGF6 regulates muscle differentiation through a calcineurin-dependent pathway in regenerating soleus of adult mice. J Cell Physiol. 2005;204(1):297-308. 15672378 10.1002/jcp.20302

[36] Pedersen BK . The anti-inflammatory effect of exercise: its role in diabetes and cardiovascular disease control. Essays Biochem. 2006;42:105-17. 17144883 10.1042/bse0420105

[37] Gentili A , Zaibi MS, Alomar SY, et al Circulating levels of the adipokines monocyte chemotactic protein-4 (MCP-4), macrophage inflammatory protein-1β (MIP-1β), and eotaxin-3 in severe obesity and following bariatric surgery. Horm Metab Res. 2016;48(12):847-853. 27300476 10.1055/s-0042-108731

[38] Pradeep AR , Kumari M, Kalra N, Priyanka N. Correlation of MCP-4 and high-sensitivity C-reactive protein as a marker of inflammation in obesity and chronic periodontitis. Cytokine. 2013;61(3):772-7. 23375121 10.1016/j.cyto.2012.12.022

[39] Bull FC , Al-Ansari SS, Biddle S, et al World Health Organization 2020 guidelines on physical activity and sedentary behaviour. Br J Sports Med. 2020;54(24):1451-1462. doi:10.1136/bjsports-2020-102955. 33239350 PMC7719906

[40] You T , Arsenis NC, Disanzo BL, Lamonte MJ. Effects of exercise training on chronic inflammation in obesity: current evidence and potential mechanisms. Sport Med. 2013;43(4):243-256. 10.1007/s40279-013-0023-3 23494259

[41] Kelly AS , Steinberger J, Olson TP, Dengel DR. In the absence of weight loss, exercise training does not improve adipokines or oxidative stress in overweight children. Metabolism. 2007;56(7):1005-9. 17570265 10.1016/j.metabol.2007.03.009

[42] Vasconcellos F , Seabra A, Cunha F, et al Health markers in obese adolescents improved by a 12-week recreational soccer program: a randomised controlled trial. J Sports Sci. 2016;34(6):564-75. 26208409 10.1080/02640414.2015.1064150

[43] Kim ES , Im J-A, Kim KC, et al Improved insulin sensitivity and adiponectin level after exercise training in obese Korean youth. Obesity. 2007;15(12):3023-3030. 18198311 10.1038/oby.2007.360

[44] Han Y , Liu Y, Zhao Z, et al Does physical activity-based intervention improve systemic proinflammatory cytokine levels in overweight or obese children and adolescents? Insights from a meta-analysis of randomized control trials. Obes Facts. 2019;12(6):653-668. 31645033 10.1159/000501970 PMC6940473

[45] Alizadeh H , Safarzade A. High intensity intermittent training induces anti-inflammatory cytokine responses and improves body composition in overweight adolescent boys. Horm Mol Biol Clin Investig. 2019;39(3). doi 10.1515/hmbci-2019-0004. 31369392

[46] Wheatley SD , Deakin TA, Arjomandkhah NC, Hollinrake PB, Reeves TE. Low carbohydrate dietary approaches for people with type 2 diabetes—a narrative review. Front Nutr. 2021;8:687658. 34336909 10.3389/fnut.2021.687658 PMC8319397

